# 

**Published:** 1888-10

**Authors:** 


CONTENTS

Vol. 35 No. 10.

PAGE.
Christian Science................217
The School Age and School Hours 220
Massage and Movement Treatment...........................220
Cremation.........................221
Rapid Cure of Whooping Cough 222
Accused of Witchcraft and Burned to Death..................223
Eating more than we need.......225
A Test of Clairvoyance..........226
Saccharine.........................226
How some Girls live..............227
Art of Infant Feeding............229
Ingenious Quackery ............232
Stimulation........................233
Manners of English Society......234
Diet in Fever......................235
Book Notices.......................236
Household..........................237
Miscellaneous.....................239

INDEX TO ADVERTISEMENTS OF HALF A PAGE AND OVER.

PAGE.
Page & Co.................... I
Yankee Blade.................. I
Stevensville Mills............ II
The Tea Cure.................. II
Volapük....................... III
Hollow Suppositories......... IV
Lactopeptine.................. V
Premiums of Books............ VI
Bovinine...................... VIII
J. Albert Kimball, D. D. S..... IX
Rio Chemical Co............... X
Davis' Elixir of Health....... XI

